# Responses of regulatory and effector T-cells to low-dose interleukin-2 differ depending on the immune environment after allogeneic stem cell transplantation

**DOI:** 10.3389/fimmu.2022.891925

**Published:** 2022-08-02

**Authors:** Yusuke Meguri, Takeru Asano, Takanori Yoshioka, Miki Iwamoto, Shuntaro Ikegawa, Hiroyuki Sugiura, Yuriko Kishi, Makoto Nakamura, Yasuhisa Sando, Takumi Kondo, Yuichi Sumii, Yoshinobu Maeda, Ken-ichi Matsuoka

**Affiliations:** Department of Hematology and Oncology, Okayama University Graduate School of Medicine, Dentistry and Pharmaceutical Sciences, Okayama, Japan

**Keywords:** regulatory T cell, low-dose interleukin-2 therapy, graft-versus-host disease, graft-versus-leukemia effect, transplantation tolerance

## Abstract

CD4^+^Foxp3^+^ regulatory T cells (Tregs) play a central role in the maintenance of immune tolerance after allogeneic hematopoietic stem cell transplantation (HSCT). Tregs promptly respond to low concentrations of IL-2 through the constitutive expression of high-affinity IL-2 receptors. It has been reported that low-dose IL-2 therapy increased circulating Tregs and improved clinical symptoms of chronic GVHD. Clinical studies of IL-2 therapy so far have mainly targeted patients in the chronic phase of transplantation when acute immune responses has subsided. However, the biological and clinical effects of exogenous IL-2 in an acute immune environment have not been well investigated. In the current study, we investigated the impact of exogenous IL-2 therapy on the post-transplant homeostasis of T cell subsets which influence the balance between GVHD and GVL in the acute phase, by setting the various immune environments early after HSCT in murine model. We initially found that 5,000 IU of IL-2 was enough to induce the active proliferation of Treg without influencing other conventional T cells (Tcons) when administered to normal mice. However, activated Tcons showed the response to the same dose of IL-2 in recipients after allogeneic HSCT. In a mild inflammatory environment within a threshold, exogenous IL-2 could effectively modulate Treg homeostasis with just limited influence to activated T cells, which resulted in an efficient GVHD suppression. In contrast, in a severely inflammatory environment, exogenous IL-2 enhanced activated T cells rather than Tregs, which resulted in the exacerbation of GVHD. Of interest, in an immune-tolerant state after transplant, exogenous IL-2 triggered effector T-cells to exert an anti-tumor effect with maintaining GVHD suppression. These data suggested that the responses of Tregs and effector T cells to exogenous IL-2 differ depending on the immune environment in the host, and the mutual balance of the response to IL-2 between T-cell subsets modulates GVHD and GVL after HSCT. Our findings may provide useful information in the optimization of IL-2 therapy, which may be personalized for each patient having different immune status.

## Introduction

Allogeneic hematopoietic stem cell transplantation (HSCT) cures hematological malignancies, however, graft-versus-host disease (GVHD) induced by allo-reactive donor T cells remains to be a major cause of morbidity after HSCT. On the other hand, allogeneic immune reaction also provides the beneficial graft-versus-leukemia (GVL) effects, therefore the preferential suppression of GVHD without sacrificing the GVL activity is an important goal for HSCT.

Regulatory T cells (Tregs) are critical to self-tolerance (1–4). In inflammatory microenvironments, activated effector T cells produce IL-2 which supports the further expansion of activated effector T cells in a positive feedback loop. Tregs promptly respond to secreted IL-2 through the constitutive expression of high-affinity IL-2 receptors, and inhibit effector T cells and suppress inflammation.

In the context of allogeneic HSCT, Tregs play a central role in controlling GVHD and inducing immune tolerance. Initial studies demonstrated that adoptively transferred Tregs prevented GVHD in mice (5, 6), and, of note, Tregs preserved the GVL activity while inhibiting GVHD (7). Interestingly, it was shown that CD62L^+^ or CCR7^+^ Tregs are more potent to suppress GVHD than CD62^-^ or CCR7^-^ counterparts presumably due to facilitating Treg to entering into the lymph nodes as priming sites of GVHD (8–10).

From the point of view of the separation of GVHD and GVL, murine and clinical studies have suggested that Interleukin-2 (IL-2) therapy has the potential to appropriately regulate post-transplant immunity (11–17). IL-2 is an essential cytokine to differentiate and maintain Tregs (18, 19). In 1990s, murine studies and clinical trials suggested that IL-2 therapy after autologous HSCT or T-cell depleted allogeneic HSCT could modulate GVHD without reducing the GVL activity (11–15). Based on these findings, a phase 1 trial of administering low-dose IL-2 daily in patients with steroid-refractory chronic GVHD was conducted (16). In the clinical trial, 12 patients of the evaluable 23 patients had a major response to chronic GVHD symptoms, and the number of Tregs was preferentially increased in all patients (16). Biological analyses revealed that low-dose IL-2 induces the selective increase of STAT5 phosphorylation in Tregs and changes in Treg homeostasis, including increased proliferation, increased thymic export, and enhanced resistance to apoptosis (17). We also demonstrated that the expression of programmed cell death 1 (PD-1) has a crucial role in modulating Treg homeostasis during low-dose IL-2 therapy (20, 21).

Under homeostatic conditions, Tregs constitutively express CD25 but not T conventional cells (Tcons). Therefore, low-dose IL-2 will selectively promote Treg proliferation. In contrast, under inflammatory conditions Tcons will also express CD25 and consequently, the effect of low-dose IL-2 could not only induce Treg expansion but also Tcons. Thus, in a context of allo-HSCT, administration of low-dose IL-2 after transplant could induce proliferation of both cell populations. Previous studies have shown the effect of low dose IL-2 on increasing the Treg levels mainly in the chronic phase after HSCT (16, 17, 20, 22–24). In the chronic phase after HSCT, the acute inflammatory environment is settling down and Tcons often have reduced expression of CD25. This allows IL-2 administration therapy to selectively stimulate Tregs that constitutively express CD25, without stimulating other conventional T cells (17, 25). On the other hand, the impact of IL-2 administration very early after HSCT has not been well studied. In the acute phase post-transplant, the host immune environment is often significantly affected by acute allogeneic responses and CD25 can be expressed on activated effector T cells as well as Tregs. Since not only Tregs but also activated alloreactive effector T cells can respond to exogenous IL-2 therapy in the acute phase early after HSCT, more complicated environmental factors can influence the *in vivo* effects of low-dose IL-2 therapy.

Here we have investigated the impact of low-dose IL-2 therapy on T cell subsets and on the balance between GVHD and GVL in the acute immune environment by using the murine BMT model.

## Results

### Low-dose IL-2 selectively stimulates Tregs both in human and mouse in the steady-state

**Figure 1:**
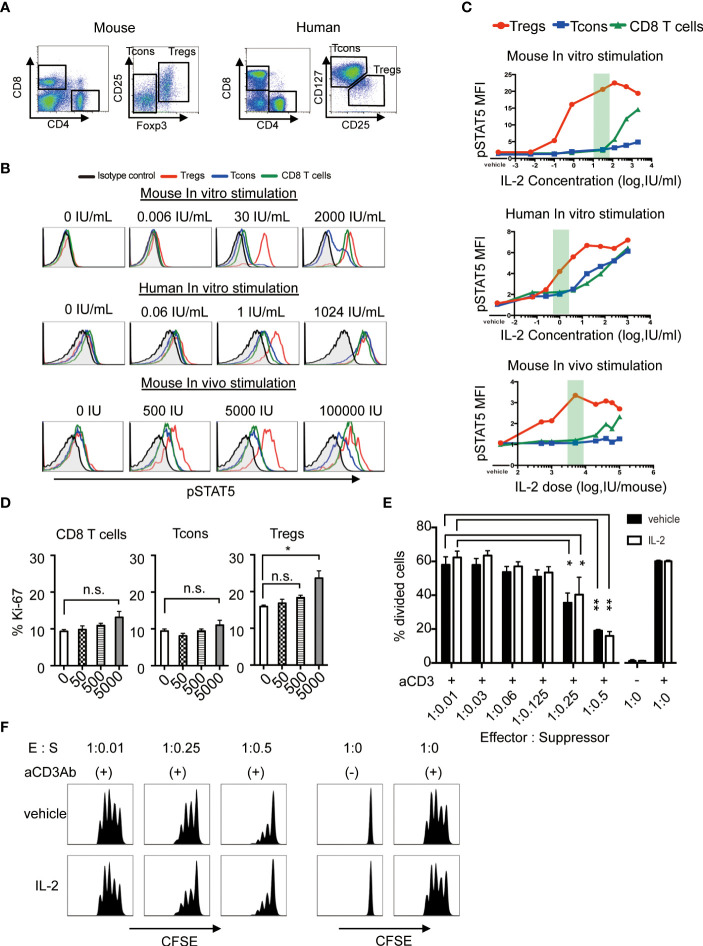
Low-dose IL-2 selectively stimulates Tregs both in human and mouse in the steady-state. **(A)** Representative lymphocyte gates for identification of CD4 and CD8 T cell subsets in mouse and human. Within the CD4 T cell gate in murine cells, Tregs are identified as CD4^+^CD25^+^Foxp3^+^ cells and Tcons are identified as CD4^+^CD25^-^Foxp3^-^ cells. Within the CD4 T cell gate in human cells, Tregs are identified as CD4^+^CD25^high^CD127^low^ cells and Tcons are identified as CD4^+^CD25^low^CD127^high^ cells. **(B)** Representative histograms of STAT5 phosphorylation and **(C)** mean fluorescence intensity of phosphorylated STAT5 in CD8 T cells (green), Tcons (blue), and Tregs (red), and from isotype control against CD4 T cells (black). Red shades indicate the dosage of low-dose IL-2 in each graph. For *in vitro* stimulation, wild type C57BL/6 mouse spleen cells and human peripheral blood mononuclear cells were stimulated with various concentrations of IL-2 for 30 minutes *in vitro*. For *in vivo* stimulation, wild type C57BL/6 mice received single doses of recombinant IL-2 and spleen cells were harvested after 30 minutes. The level of intracellular pSTAT5 was determined by flow cytometry. **(D)** Wild type C57BL/6 mice received 50, 500 and 5000 IU of recombinant IL-2 once daily for 7 days and spleen cells were analyzed assessed Ki-67 expressions in CD8 T cells, Tcons and Tregs. **(E, F)** *In vitro* Treg suppression assay. Tcons labeled with CellTrace™ Violet were cultured with Tregs isolated from mice treated with vehicle or 5000 IU of IL-2 in the presence of antiCD3 antibody stimulation for 3 days. **(E)** Percentage of divided Tcons at various Tcon : Treg cell ratios. Responder Tcons (1×10^5^ per each well) were cultured with various numbers of suppressor Tregs. n = 3 mice per group per experiment. *p <0.05**, *p <*0.005. **(F)** Representative flow cytometry histograms measuring Tcon proliferation in the presence or absence of Tregs. Data are representative of at least two independent experiments **(A–F)**. ns, not significant.

Murine and human Tregs were defined as CD4^+^CD25^high^Foxp3^+^ and CD4^+^CD25^high^CD127^low^ cells, respectively (**Figure 1A**). The levels of STAT5 phosphorylation in each CD8^+^ T, CD4^+^ Tcons and Treg subset after *in vitro* or *in vivo* exposure with various concentrations of IL-2 was assessed by flow cytometry. As shown in the top panels of **Figures 1B, C**, high concentrations of IL-2 induced STAT5 phosphorylation in all murine T cell subsets, but lower concentrations of IL-2 induced STAT5 phosphorylation only in Tregs. Similar results were obtained in human lymphocytes (the middle panels of **Figures 1B, C**). We also showed that single injection of 5,000 IU of IL-2 into normal C57BL/6 (B6) mice optimally induced STAT5 phosphorylation selectively in Tregs, but not in Tcons and CD8 T cells (the bottom panels of **Figures 1B, C**).

Then, we treated mice by daily administration of various doses of IL-2 for 7 days, and assessed the cell proliferation by the expression of Ki-67 in each T cell subset. The results showed that 5,000 IU of IL-2 is enough to induce the active proliferation of Treg without influencing other CD8^+^ T cells and CD4 Tcons (**Figure 1D**). To assess whether the proliferated Tregs could maintain their suppressive activity, we performed *in vitro* suppression assay and confirmed that Tregs from IL2-treated mice maintained the equivalent suppressive function as compared to Tregs from control mice (**Figures 1E, F**). These results suggest that this dose of IL-2 could be appropriate for Treg-oriented immune therapy models those are relevant to clinical low-dose IL-2 therapy.

### Effect of low-dose IL-2 on *in-vivo* proliferation of T cell subsets

To evaluate the effects of exogenous IL-2 on T cell subsets homeostasis *in vivo* in each syngeneic or allogeneic environment, we next performed the adoptive transfer experiments. We stained CD45.1^+^ B6 spleen cells with cell trace violet-dye and adoptively transferred them into irradiated CD45.2^+^ B6 syngeneic recipient mice or irradiated B6D2F1 allogeneic recipient mice. Thereafter, we administrated 5000 IU of IL-2 or vehicle once a day for 5 days and assessed the cell proliferation of donor-type CD8 T cells, Tcons and Tregs by the dilution of violet dye. Expression levels of CD62L on each subset were also examined.

**Figure 2:**
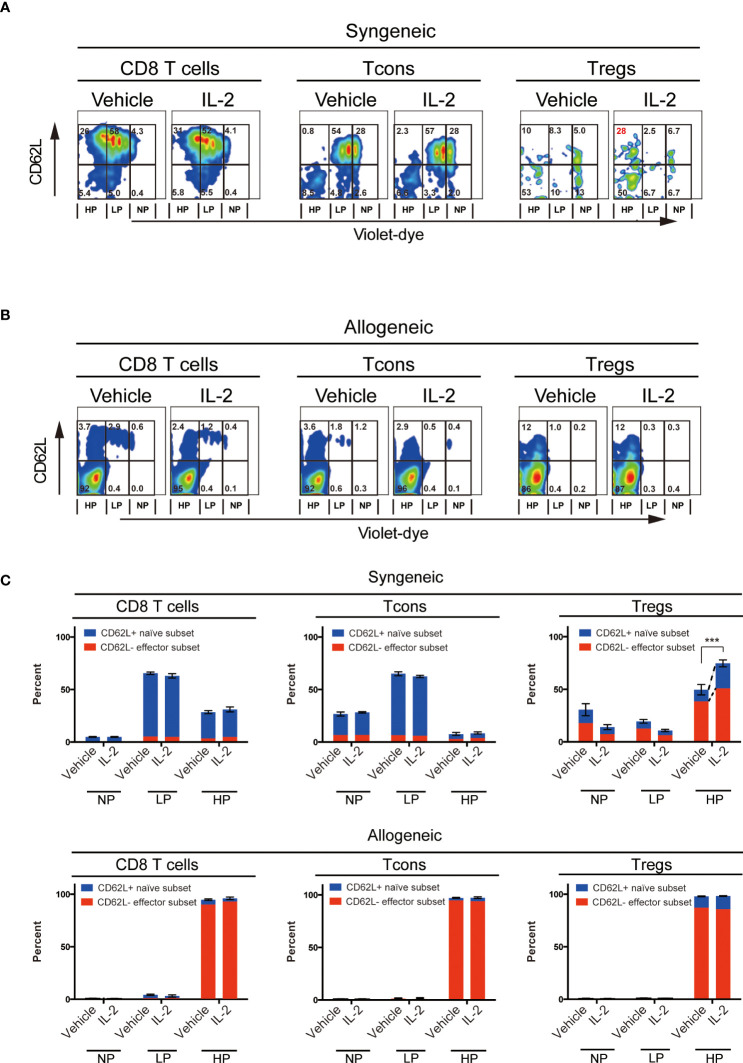
Effect of Low-dose IL-2 on *in-vivo* proliferation and CD62L expression of T cells. Spleen cells of B6 mice or B6D2F1 mice stained with CellTrace™Violet, were transplanted into irradiated (10 Gy) B6 recipient mice and recipient mice were treated with IL-2 for 5 days. (n=5) **(A, B)** Representative figures of the expression of CD62L and the dilution of CellTrace™Violet in CD8 T cells, CD4^+^ Tcons and CD4^+^CD25^+^Foxp3^+^Tregs. **(A)** shows syngeneic settings, in which setting, spleen cells of B6 mice were transplanted and **(B)** shows allogeneic settings, in which setting spleen cells of B6D2F1 mice were transplanted. Cells were grouped into 3 populations; those are NP (; no-proliferating cells), LP (; Low proliferating cells) and HP (; High proliferating cells). Cells in NP are those did not divide. Cells in LP are those divided 2 to 5 times. Cells in HP are those divided more than 6 times. **(C)** Bars show mean percent +/- SEM of NP, LP and HP cells of each T cell subset. ***p <0.005. Blue means the CD62L^+^ naïve subset and red means the CD62L^-^ effector subset, respectively. Data shows the representative result from two individual experiments.

The syngeneic transfer experiment showed that the short-term administration of IL-2 had initiated selective Treg increase (**Figures 2A, C**). Highly proliferated Tregs after IL-2 treatment maintained significantly more proportions of CD62L^+^ cells than vehicle-treated group (**Figure 2C** upper panel). CD8 T cells and Tcons did not show any difference between vehicle and IL-2 treated groups. These data suggest that the effects of low-dose IL-2 therapy on cell proliferation and CD62L expression in Treg is clearly distinct to other CD8 T cells and Tcons.

In contrast, the allogeneic transfer experiment showed that all T cell subsets, including Treg, aggressively proliferated and lost CD62L expression in both vehicle- or IL-2-treated groups.

### Effect of low-dose IL-2 on T cell activation and proliferation in the allogeneic system

**Figure 3:**
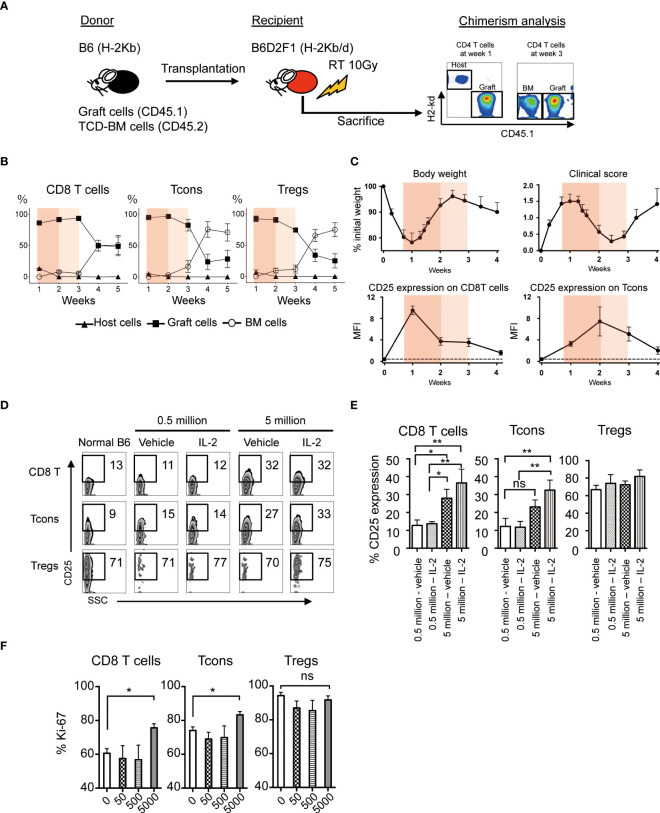
Effect of Low-dose IL-2 on T cell activation and proliferation in the allogeneic system. **(A-C)** Lethally irradiated (10 Gy) B6D2F1 mice received 5×10^6^ CD45.2^+^ TCD-BM and 5×10^6^ CD45.1^+^ spleen cells from B6 donor mice. Post-transplant treatments with IL-2 or vehicle were not administrated in this experiment. **(A)** Representative figures of chimerism analysis at week 1 and 3 are shown. Graft-derived cells, BM-derived cells and host-residual cells were defined as CD45.1^+^H-2Kd^-^, CD45.1^-^H-2Kd^-^ and CD45.1^-^H-2Kd^+^, respectively. **(B)** % Host- (closed triangle), graft- (closed square) or BM- (open circle) derived cells of CD8 T cells, Tcons and Tregs from week 1 to 5 are shown. Bar graphs are means +/- SEM. **(C)** Body weight, clinical GVHD score, MFI of CD25 expression of CD8 T cells and Tcons from week 0 to week 4 after transplantation. Thin and thick orange shade indicates the two different phases of clinical GVHD. The dash line represents the baseline at week 0. **(D, E)** Lethally irradiated B6D2F1 recipient mice were transplanted 5×10^5^ or 5×10^6^ spleen cells and 5.0×10^6^ TCD-BM from donor B6. Recipients were treated with 5000 IU IL-2 for 7 days and CD25 expressions of each lymphocyte were examined. **(D)** Representative figures of CD25 expressions on CD8 T cells, Tcons and Tregs. **(E)** Bars show mean % CD25 expression +/- SEM are shown. *p < 0.05, **p < 0.005. **(F)** 50, 500 and 5000 IU of IL-2 or vehicle were administered subcutaneously into recipient mice after BMT for 7 days and assessed Ki-67 expressions in CD8 T cells, Tcons and Tregs. Bars show mean MFI of Ki-67 +/- SEM. *p < 0.05, **p < 0.005. ns, not significant.

To evaluate the effect of low-dose IL-2 on the outcome of allogeneic HSCT, we established the murine model, which reflects clinical low-dose IL-2 therapy for patients with GVHD. Spleen cells from CD45.1^+^ B6 mice together with T-cell-depleted bone marrow (TCD-BM) from CD45.2^+^ B6 mice were transplanted to lethally irradiated B6D2F1 recipient mice and low-dose IL-2 were administrated once a day from day5 to day20 (**Figure 3A**). Chimerism and cell activation of each T cell subset in spleen was evaluated weekly for the first month after BMT.

First, we checked the chimerism of each T cell subset in the experimental HSCT system. The data demonstrated that most of T cells in the first 3 weeks after BMT were derived from mature T cells involved in the donor graft. Thereafter, the graft-derived cells were replaced by bone marrow derived cells after 4 weeks of BMT (**Figure 3B**).

Then we examined the body weight, clinical GVHD score, and CD25 expression on CD8 T cells and Tcons (**Figure 3C**, **Supplemental Figure S3**). CD25 is the IL-2 receptor alpha and reflects cell activation. GVHD score peaked at week 1 with body weight loss, and then gradually recovered until week 3. The expression of CD25 on CD8 T cells and Tcons was increased during weeks 1 and 2, and decreased thereafter. These results suggest that the first 3 weeks, when the majority of each T cell subset is derived from graft cells, could be further subdivided into 2 phases; the phase of acute immune responses in the early after transplantation (week 1 to 2, painted by deep orange) and the following phase of recovering from the aggressive immune environment (week 2 to 3, painted by light orange) (**Figures 3B,C**). To assess cell activation and proliferation of CD8 T cells and Tcons by IL-2 treatment immediately after transplant, we administered 5000 IU IL-2 for 7 days from the day of BMT (**Figures 3D, E**). When 0.5 million allogeneic spleen cells were transplanted and 5000 IU of IL-2 was daily administered to the recipients, IL-2 did not increase CD25 expression on CD8 T cells and Tcons (**Figures 3D, E**). In contrast, when 5 million allogeneic spleen cells were transplanted and IL-2 was daily administered, IL-2 significantly increased CD25 expression on these cells (**Figures 3D, E**). However, IL-2 treatment with further reduced doses did not promote cell proliferation of CD8 T cells and Tcons even after the transplantation of 5 million allogeneic spleen cells (**Figure 3F**). On the other hand, Tregs showed very active proliferation in the acute phase after HSCT even without IL-2 therapy (**Figure 3F**). These data demonstrated that IL-2 administration within the first week immediately following HSCT may enhance the activity of CD8 T cells and Tcon without affecting Treg homeostasis, thus suggesting it could have the opposite effect on tolerance induction.

### Effect of low-dose IL-2 on T cell differentiation status in the allogeneic system

**Figure 4:**
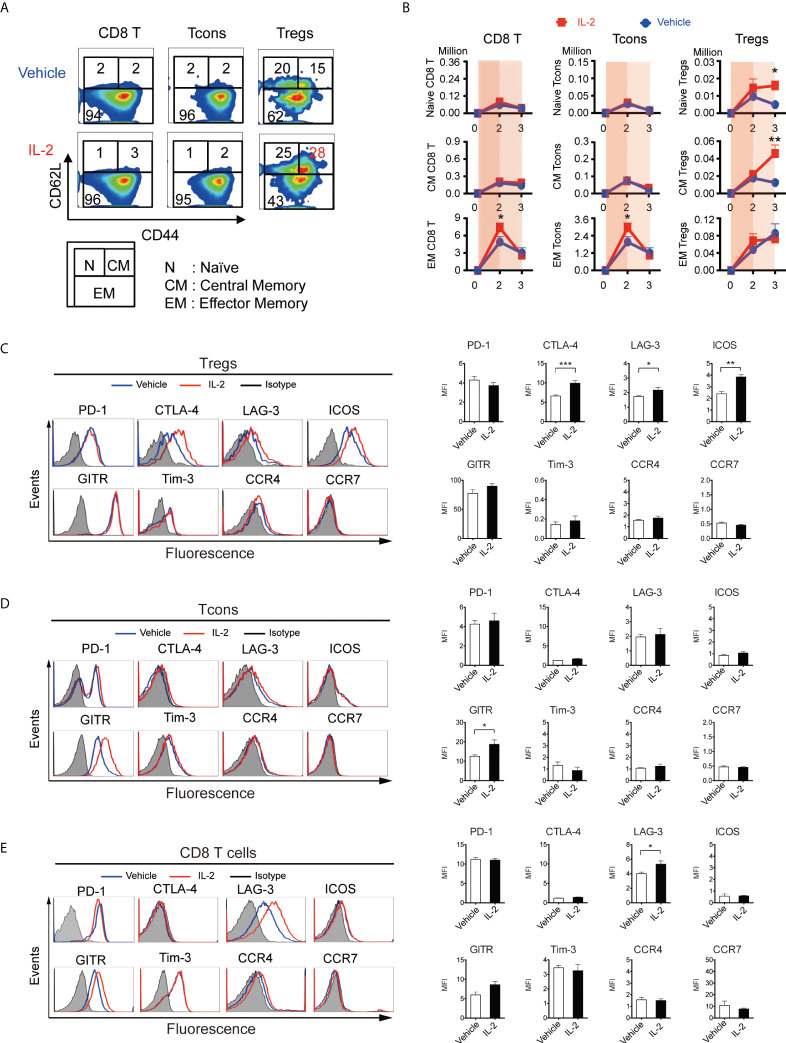
IL-2 therapy provides different effects depending on the immune environment when it is administered. **(A-D)** Lethally irradiated (10 Gy) B6D2F1 mice were transplanted with 5 × 10^6^ spleen cells and 5 × 10^6^ bone marrow cells from donor B6 mice, and vehicle or 5000 IU of IL-2 were subcutaneously administrated once per day for 15 days. Spleen cells were analyzed at week2 and week3 after transplantation. **(A)** Representative figures to identify CD44^low^CD62L^high^ naive (N), CD44^high^CD62L^high^ central-memory (CM), and CD44^high^CD62L^low^ effector-memory (EM) subsets within CD8 T cells, Tcons, and Tregs at week 3. Upper and lower panels are representative of mice treated with vehicle and IL-2, respectively. **(B)** Absolute number of naïve, central-memory, and effector-memory subsets in CD8 T cells, Tcons, and Tregs in mice treated with vehicle (blue) or IL-2 (red). Thin and thick blue shade indicates the two different phases based on Figure 3C. Data shows mean +/- SEM. *, *p* < 0.05; **, *p* < 0.005 vs. vehicle. **(C-E)** Representative histograms and mean fluorescence intensity of PD-1, CTLA-4, LAG-3, ICOS, Tim-3, GITR, Tim-3, CCR4 and CCR7 expression in **(C)** Tregs, **(D)** Tcons and **(E)** CD8 T cells following treatment with control vehicle or IL-2 at week 3. Data are mean ± SEM. *, *p* < 0.05; **, *p* < 005; ***, *p* < 0.0005.

Based on the above findings, we scheduled 5000 IU IL-2 administration in this experiment from Day 5 (week 1) to Day 20 (week 3), avoiding the 4 days immediately following BMT. First, to assess the homeostasis of T cell subsets through the first 3 weeks after allogeneic transplant, we evaluated the expression of differentiation markers, inhibitory co-stimulating molecules, and chemokine receptors (**Figures 4A-D**). Lethally irradiated B6D2F1 mice were transplanted with CD45.1^+^ 5 × 10^6^ spleen cells and 5 × 10^6^ TCD-BM from B6 mice, treated with vehicle or IL-2 from day5, and sacrificed at day 14 (week 2) and day 21 (week 3) for analyses. Our data demonstrated that CD8 T cells and Tcons sharply expanded with CD44^+^CD62L^-^ effector/memory phenotype in IL2-treated mice at day 14 (**Figures 4B**, **Supplemental Figure S2**). However, the extended IL-2 administration resulted in the contraction of these effector T cells at day 21 (week 3). In contrast, Tregs expanded with CD44^-^CD62L^+^ naïve- or CD44^+^CD62L^+^ central/memory-phenotypes in IL2-treated mice at day 21 (**Figures 4A, B**).

We quantified the expression of co-stimulatory molecules as well as migration markers on Tregs at week 3 (**Figure 4C**). Of these, CTLA-4 (mean fluorescence intensity 6.61 vs 9.92, p < 0.001), LAG-3 (1.73 vs 2.17, p = 0.04), and ICOS (2.41 vs 3.84, p < 0.001) were up-regulated with IL-2 therapy, although the expression of chemokine receptor CCR4 and CCR7 did not change. We also quantified the expression of them on Tcons and CD8 T cells. GITR (12.5 vs 18.7, p = 0.02) on Tcons was up-regulated with IL-2 therapy, which was not up-regulated on Tregs. LAG-3 on CD8 T cells (4.01 vs 5.30, p = 0.02) was also up-regulated, as it on Tregs.

### IL-2 therapy provides different effects depending on the immune environment when it is administered

**Figure 5:**
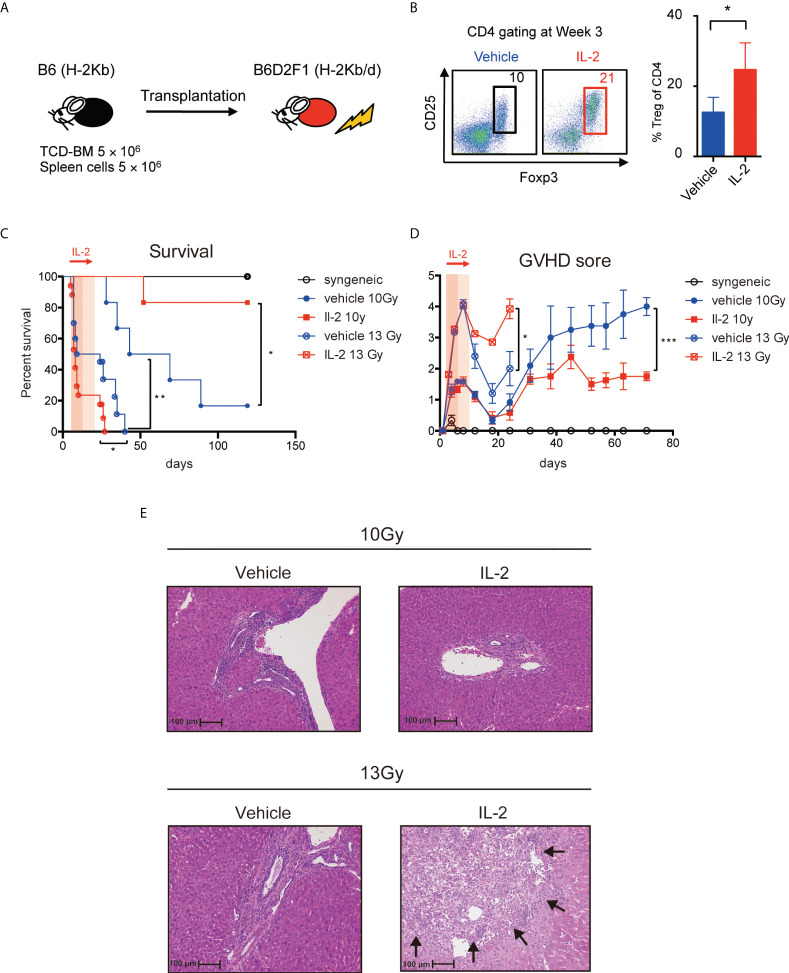
IL-2 has different clinical impact on GVHD by the conditioning of the transplantation. **(A)** Lethally irradiated (10 Gy or 13 Gy) B6D2F1 mice received 5 × 10^6^ TCD-BM and 5 × 10^6^ spleen cells from B6 donor mice. Vehicle or 5000 IU IL-2 was subcutaneously administrated once per day from day 5 to day 20. **(B)** Percentages of Tregs at week 3 after transplantation are shown. Dot plots are average ± SEM. *, *p* < 0.05. **(C)** Survival rates. *, *p* < 0.05; **, *p* < 0.005. **(D)** Clinical GVHD scores. *, *p* < 0.05; ***, *p* < 0.0005. **(E)** The representative pathological findings in liver histology at week 5 are shown. The necrotic foci are pointed with arrows.

To assess the clinical effect of low-dose IL-2 therapy, we treated the lethal GVHD preclinical model, in which model B6D2F1 mice were lethally irradiated and transplanted with 5 million spleen cells and 5 million bone marrow cells from B6 mice and then 5000 IU of IL-2 was administered day 5 to day20 (**Figure 5A**). CD4^+^Foxp3^+^ Tregs, measured as % of all CD4^+^ T cells, were more abundant at day 21 in animals treated with IL-2 (**Figure 5B**). To compare the clinical impact of IL-2 therapy in the different inflammatory conditions after BMT, two different irradiation settings (10 Gy and 13 Gy) were prepared. Recipients irradiated with 13 Gy showed the significantly shorter survival with the higher GVHD score than those irradiated 10 Gy in both vehicle treated and IL-2 treated groups. When recipient mice were irradiated 10 Gy, survival was significantly longer in mice treated with IL-2 than in mice treated with vehicle (**Figure 5C**). The latter developed severe GVHD with significantly higher clinical scores than IL-2-treated recipients from day 35 and thereafter (**Figure 5D**). In contrast, when recipient mice were irradiated 13 Gy, the early developed GVHD showed higher mortality with more severe GVHD scores in IL-2 treated recipients than vehicle treated recipients (**Figures 5C, D**). The representative pathological findings comparing between these arms at week 5 are observed in liver histology (**Figure 5E**). In 10 Gy setting, more infiltration of lymphocytes and neutrophils were observed in vehicle-treated mice than IL-2-treated mice. In 13 Gy setting, the cell infiltration was observed in both groups but larger necrotic foci and bile duct injury were observed in IL-2-treated mice. These suggest that IL-2 may provide opposite effects depending on the immune environment when it is administered. IL-2 therapy ameliorates clinical GVHD in the mild inflammatory state but it may even exacerbate GVHD in the intense inflammatory state.

### IL-2 therapy ameliorates clinical GVHD without sacrificing the GVL activity in the mild inflammatory state

**Figure 6:**
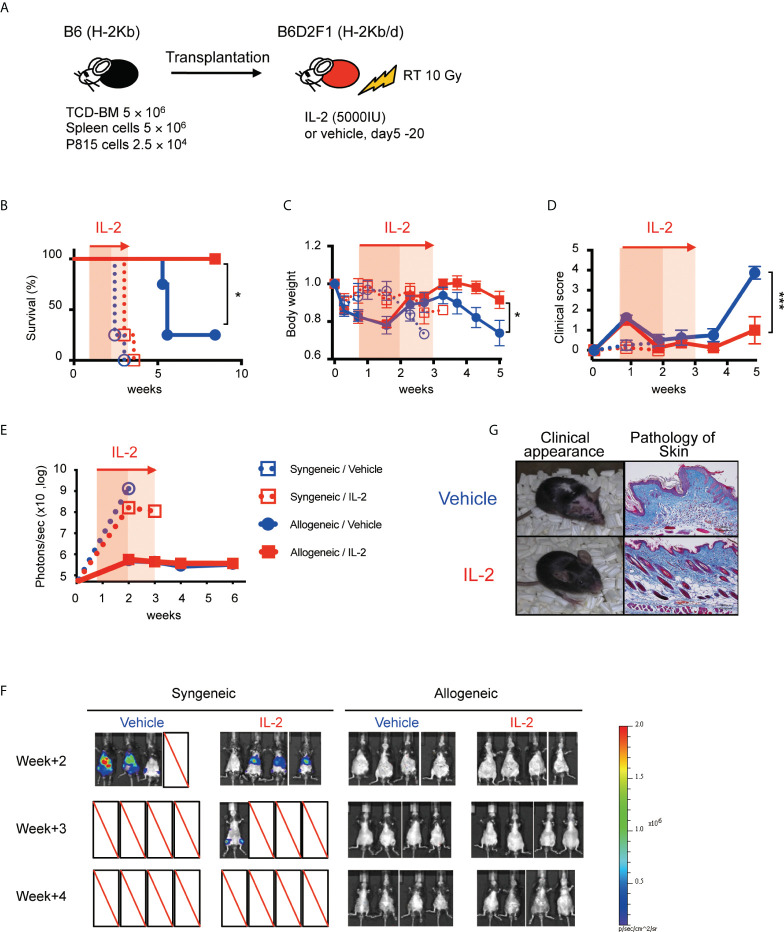
IL-2 therapy ameliorates clinical GVHD without sacrificing the GVL activity in the mild inflammatory state. **(A)** Lethally irradiated (10 Gy) B6D2F1 mice received 5 × 10^6^ TCD-BM and 5 × 10^6^ spleen cells from B6 donor mice, together with or without 2.5 × 10^4^ luciferase^+^ P815 tumor cells. Vehicle or 5000 IU IL-2 were subcutaneously administrated once per day from day 5 to day 20. **(B–E)** Survival rates, body weight, clinical GVHD scores and bioluminescence after transplant were shown. Mean +/- SEM of *, *p* < 0.05; ***, *p* < 0.0005. **(F)** Bioluminescent signals of P815 tumors in 4 experimental groups are shown. **(G)** Appearance and skin pathology at week 5 in representative mice treated with vehicle and IL-2.

To assess the clinical effect of IL-2 therapy for recipients with active tumor, recipient B6D2F1 mice were irradiated with 10 Gy, and transplanted with 5 × 10^6^ spleen cells and 5 × 10^6^ TCD-BM from B6 (syngeneic) or B6D2F1 (allogeneic) mice, along with 2.5 × 10^4^ P815 (H-2Kd) cells expressing luciferase (**Figure 6A**). IL-2 or control vehicle were then administered from day 5 to day 20, and monitored body weight, clinical GVHD score, and survival. Tumor burden in each mouse was also quantified weekly by bioluminescence imaging as described in Methods.

All syngeneic group mice died by tumor before week 4 and allogeneic transplanted mice survived significantly longer (**Figure 6B**). Bioluminescence imaging study demonstrated that syngeneic group mice had high tumor burden at week 2 while allogeneic group mice appeared to clear tumor cells (**Figures 6E, F**). Density of photons was equivalent between IL2-treated allo-recipients and control allo-recipients, suggesting IL-2 therapy did not sacrifice the GVL activity. Allogeneic transplanted mice treated with IL-2 had significantly lower GVHD scores and better recovery of body weight than those with treated with control vehicle (**Figures 6C, D, G**).

### IL-2 therapy enhances the GVL activity without causing clinical GVHD in the immune-tolerant state

**Figure 7:**
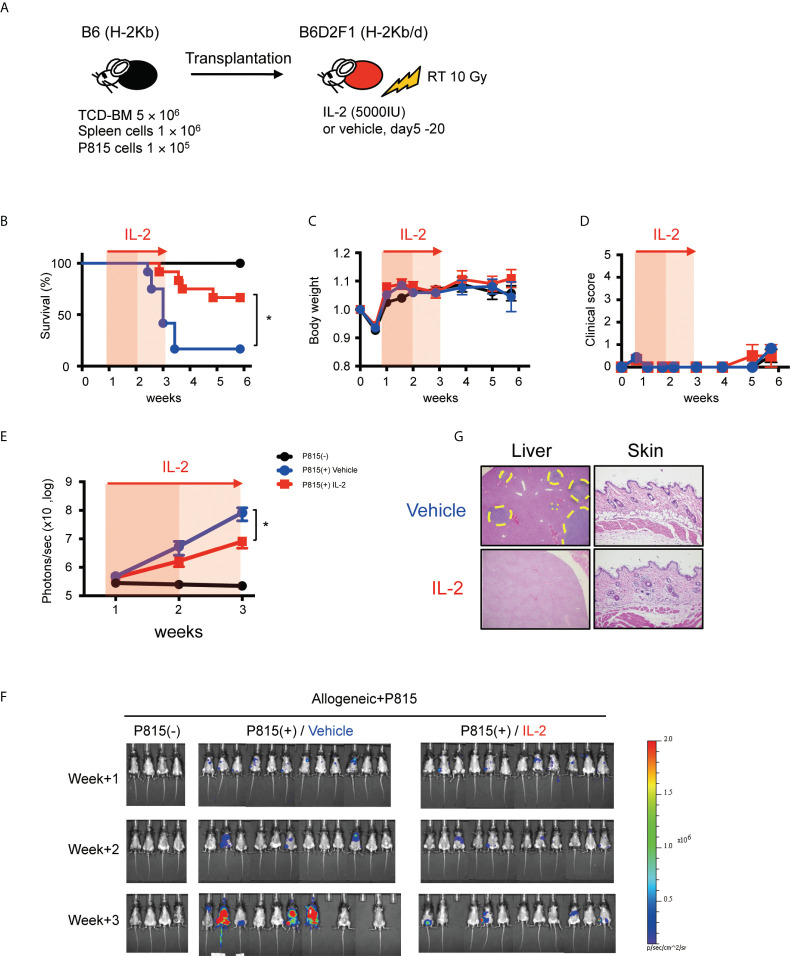
IL-2 therapy enhances the GVL activity without causing clinical GVHD in the immune-tolerant state. **(A)** Lethally irradiated (10 Gy) B6D2F1 mice received 5 × 10^6^ TCD-BM and 1 × 10^6^ spleen cells from B6 donor mice, together with or without 1 × 10^5^ luciferase^+^ P815 tumor cells. Vehicle or 5000 IU IL-2 were subcutaneously administrated once per day from day 5 to day 20. **(B–E)** Survival rates, body weight, clinical GVHD scores and bioluminescence after transplant were shown. Mean +/- SEM of *, *p* < 0.05 **(F)** Representative bioluminescence images of P815 tumors are shown. **(G)** Liver and skin pathology at week 3 in mice treated with vehicle and IL-2 are shown. Yellow dot circles indicate tumor occupying lesions.

To evaluate the impact of IL-2 on the GVL activity more precisely, we tested the anti-tumor effect in less inflammatory transplant setting with reduced splenocytes (1×10^6^) and increased tumor cells (1×10^5^), because this experimental setting minimizes the impact of GVHD on survival and can focus primarily on the GVL activity (**Figure 7A**). In fact, in this transplant setting, recipient mice did not show the significant features of clinical or pathological signs of GVHD, irrespective of IL-2 treatment (**Figures 7C, D, G**). Bioluminescence imaging study demonstrated that most of IL2-treated allo-recipients appeared to clear tumor cells while half of control allo-recipients still bore tumor cells at week 3 (**Figures 7F, G**). Density of photons was significantly lower in IL2-treated allo-recipients than in control allo-recipients (**Figure 7E**). These data indicate that IL-2 therapy may enhance the GVL activity without exacerbating GVHD in the immune-tolerant state.

## Discussion

Tregs promptly respond to low concentrations of IL-2 through the constitutive expression of high-affinity IL-2 receptors. In contrast, conventional T cells (Tcons) do not express CD25 in the steady-state, however, they also express CD25 after activation and become responsive to endogenous and exogenous IL-2 in the inflammatory environment. The experimental and clinical studies of IL-2 therapy so far have mainly targeted normal mice in a steady-state or patients in the chronic phase of transplantation, and therefore the effects of exogenous IL-2 on the acute phase when acute immune responses are ongoing have not been fully investigated. In the current study, we demonstrated that the balance between responses of Tregs and effector T cells to exogenous IL-2 differ according to the activity of the immune state in the host. The biological effects of exogenous IL-2 on each T subsets modulate GVHD and GVL in recipients after HSCT. Our results may provide useful information in the optimization of IL-2 therapy, which may be personalized for each patient having different immune status.

First, we performed the titration of IL-2 dosage to find the suitable dose that selectively activates Tregs but not CD8 T cells or Tcons (**Figure 1**). In both murine and human lymphocytes, we confirmed that a high dose of IL-2 stimulated not only Tregs but also other conventional T cells, in contrast, lower doses of IL-2 could stimulate Tregs selectively (**Figures 1B, C**). By administering different doses of IL-2 to mice in the steady-state, we checked the effect of IL-2 less than 5000 IU on cell proliferation and found 5000 IU was appropriate (**Figure 1D**). *In vivo* IL-2-expanded Treg showed the equivalent potential to suppress effector T cells *in vitro* (**Figures 1E, F**).

Next, to consider the impact of the immune environment of recipients on cell proliferation after 5000 IU IL-2 administration, we performed the adoptive transfer experiment (**Figure 2**). When lymphocytes were transferred to syngeneic recipients followed by IL-2 administration, IL-2 promoted Treg proliferation as compared to vehicle treatment. In contrast, when lymphocytes were transferred to allogeneic recipients followed by IL-2 administration, the benefit of IL2 administration was abrogated when administered to mice with intense allogeneic stimulation. This clearly indicated that not only the dosage of IL-2 but also the *in vivo* immune environment of the recipient when IL-2 was administered is important to obtain the expected immune response by IL-2 therapy.

Then, considering the case that IL-2 is administered to recipients under the intense allogeneic environment, we investigated changes in the clinical and immune status of recipients in the acute phase after allogeneic HSCT (**Figures 3A-C**). In the first week, each T cell subset including Treg contained the host-derived residual population and those disappeared in week 2 (**Figures 3B**, **Supplemental Figure S1**). Main compartments of each lymphocyte were derived from mature cells in the graft during the first 3 weeks, and newly-differentiated lymphocytes from bone marrow emerged from week 4 (**Figure 3B**). During the period, clinical GVHD severity and effector T activation was peaked 1 week after HSCT and contracted thereafter (**Figure 3C**, **Supplemental Figure S3**). To investigate the possibility of IL-2 administration in the first week when the intensity of the allogeneic environment peaks, we initiated IL-2 administration just after HSCT under various experimental conditions with different infusion cell numbers and IL-2 doses (**Figures 3D-F**). The results demonstrated that 5000IU IL-2 administration to mice early after receiving 5 million allogeneic spleen cells increased CD25 expression on CD8 T cells and Tcon cells, and promoted their proliferation. This suggests that IL-2 therapy soon after HSCT could further enhance aggressive effector T cell immunity. On the other hand, CD25 expression and proliferation of Tregs were almost not influenced by IL-2 therapy in this phase. The immune microenvironment is considered to be filled with abundant IL-2 produced by activated donor T cells in addition to other inflammatory cytokines soon after HSCT. Our data suggest that Treg proliferation has already ridden to the limit in response to the increased endogenous IL-2 and there was no room for exogenously-administered IL-2 to further increase Treg proliferation. Collectively, these data indicate the possibility that IL-2 therapy very early after HSCT has the opposite effect of its original purpose of suppressing GVHD.

As shown in **Figure 3B** and **Supplemental Figure S1**, we observed that host-type residual Tregs remained on week 1 and they almost disappeared on week 2. Previous studies demonstrated that Tregs are more resistant to irradiation than conventional T cells and thus host-type residual Tregs can survive and expand after allogeneic HSCT with TBI-based conditioning (26, 27). Importantly, it has been also shown that exogenous IL-2 therapy soon after HSCT can promote the expansion of host-type Tregs which contributes to the suppression of donor effector T cells (28). These studies suggest the effect of IL-2 therapy on the homeostasis of host-type Treg soon after HSCT. Further studies to address the role of host Tregs in IL-2 therapy in the acute phase are warranted.

Based on the finding in **Figure 3**, we administered IL-2 to the allogeneic HSCT recipients from day 5 to day 20, avoiding the very acute days after HSCT, and analyzed the lymphocyte recovery in detail (**Figure 4**). IL-2 administration up to the second week, when the background immune environment was still intense, promoted expansion of effector-memory CD4 and CD8 CTLs, but did not significantly affect the number of Tregs. Extended IL-2 administration after the second week, when immune intensity has decreased, promoted the expansion of naive and central-memory Tregs and the contraction of CTLs (**Figures 4A, B**). Phenotypically, expanded Tregs highly expressed PD-1 and also increased the expression of the other suppressive immune checkpoints by IL-2 administration (**Figures 4C-E**). These data again suggest that the background immune condition has major impacts on the *in vivo* effects of exogenous IL-2. In our previous study, we reported that PD-1 has a critical role to modulate Treg homeostasis during IL-2 therapy by using a non-transplant model (20). In the current study using the allo-HSCT model, PD-1 expression on Treg appeared to already rise to the limit with or without IL-2, and IL-2 administration did not further increase the expression. However, the expression of other inhibitory molecules such as CTLA-4 and ICOS were significantly increased in the IL-2 group, suggesting the multiple inhibitory molecules might be involved in the regulation of Treg homeostasis in IL-2 therapy for inflammatory hosts after allo-HSCT.

We previously demonstrated that IL-2 administration increased CD62L^+^ central-memory Tregs by using a non-transplant model (20). The data from the current study showed the IL-2 administration also resulted in the increase of same Treg subpopulation in an allo-HSCT model as well (**Figures 4A, B**). In general, CD62L plays important role in lymphocyte migration to second lymphoid organs. In allogeneic HSCT, CD62L^+^CCR7^+^ naive T cells, but not CD62L^-^CCR7^-^ memory T cells, migrate to lymph nodes, interact with antigen-presenting cells there, and are activated and converted to allo-reactive CD62L^-^CCR7^-^ effector T cells (29–33). On the contrary, CD62L naive or central-memory Tregs enter to lymph nodes from periphery or inflammatory sites, and inhibit the priming of effector T cells (8–10, 34). Indeed, CD62L Tregs in allografts were found to be associated with lower GHVD risk in a clinical trial (35). Our data suggest that IL-2 therapy may not only increase the number of Tregs, but also alter the dynamics of migration, thereby promoting GVHD suppression.

Then, we investigated how the biological effects of exogenous IL-2 on lymphocyte subsets in the acute phase, which were shown in **Figures 2**-**4**, lead to overall survival after HSCT (**Figure 5**). In general, higher doses of TBI can cause extensive damage and activation in host tissues, which release inflammatory cytokines and enhance recipient MHC antigens, leading to the increased risk of acute GVHD (36). To examine the impact of the immune environment in IL-2 therapy soon after HSCT, we conducted HSCT with the two different TBI settings. Our data showed that IL-2 therapy provided opposite effects depending on the immune environment caused by different TBI doses (**Figures 5C-E**). It is considered that intense inflammatory condition induced by high doses of TBI (13 Gy) could increase IL-2-induced effector T cell (Teff) expansion to the unallowable levels during the first 2 weeks after HSCT (**Figures 5C, D**). In contrast, mild inflammatory condition by lower doses of TBI (10 Gy) may not promote the IL-2-induced Teff expansion and lead to an improvement in overall survival through subsequent Treg expansion (**Figures 5C, D**). Histopathological findings also indicated that IL-2 appeared to suppress infiltration of effector T cells to the target tissue at low dose settings, whereas IL-2 promoted tissue damage at high dose settings (**Figure 5E**). These data again suggest that the host immune environment has important role for the immune modulation by IL-2 therapy early after HSCT.

To consider the effect of IL-2 therapy on the GVL activity, we conducted HSCT for the recipients bearing cancer cells (**Figure 6**). Our data demonstrated that IL-2 therapy suppressed GVHD and improved the overall survival without the evidence of cancer growth until week 6 (**Figures 6E, F**), suggesting that IL-2-expanded Treg under mild inflammatory conditions did not interfere with the GVL activity.

Lastly, we examined the role of IL-2 therapy in the immune-tolerant state after HSCT (**Figure 7**). We performed HSCT with reduced graft cells and reduced TBI, which resulted in status without any clinical and pathological GVHD. IL-2 therapy prevented tumor progression, presumably due to the early expansion of effector T cells promoted by IL-2. Previously, it was reported that the prolonged infusion of low-dose IL-2 after HSCT could suppress tumor relapse without developing GVHD (14). Patients included in the clinical study were after autologous and T cell-depleted allogeneic bone marrow transplantation and did not have ongoing GVHD at the start of IL-2 therapy, thus it is thought that the patients were in the immune-tolerant state. Our murine data in the current study appear to be consistent with the previous clinical observation. Recent advances in T-cell-depletion therapy such as post-transplant cyclophosphamide have made it possible to maintain a long-term immune stable state, but on the other hand, the development of a GVL initiator for cases having a high risk of relapse is expected (37–39). IL-2 may have the potential for such roles, depending on the immune status of patients.

Collectively, the data of the current study suggests that IL-2 therapy after allogeneic HSCT could provide various clinical effects depending on the immune status of the host. In a mild inflammatory environment that endogenous IL-2 is thought to be not sufficient for Treg to be fully activated, exogenous IL-2 can effectively modulate Treg homeostasis to exert an efficient suppressive function. In an immune-tolerant state that endogenous IL-2 is thought to be not sufficient for effector T cells to be fully activated, exogenous IL-2 can trigger Teff to exert an anti-tumor effect. However, in a severely inflammatory environment that is already filled with abundant endogenous IL-2, such as immediately after transplant, it appears to be hard to find merits administering IL-2 in terms of both GVHD suppression and GVL enhancement. These results mean that we need to pay attention to the immune status of the host in the clinical IL-2 therapy. Of note, the levels of T cell activation after HSCT have been changing over time and thus the biological effects of exogenous IL-2 have been changed even during receiving IL-2 therapy.

The differences in the kinetics of CD25 expression between Tcon and Treg early after HSCT appears to be important to interpreting the various clinical effects resulting from exogenous IL-2 therapy. CD4^+^ and CD8^+^ Tcons can be distinguished from Tregs by the expression of Foxp3, CD25, and CD127 (1, 4, 40). Tregs constitutively express CD25, IL-2 receptor alpha, and are maintained by the physiological concentration of IL-2 in the steady-state (18). CD25 expression on Tregs was maintained at the high levels through the acute phase after HSCT in the current study (data not shown). In contrast to Tregs, resting Tcons do not express CD25 in the steady-state and increase the expression after cell activation. The activation of Tcons is maximized by autocrine IL-2/IL-2 receptor signaling. Therefore, the differences in CD25 expression between Tregs and Tcons can significantly alter from acute to chronic phases after HSCT, leading to the various clinical effects of IL-2 therapy.

The concept that the effect of immunotherapy could depend on the immune environment of the host is also shown in immune checkpoint blockade therapy. A recent study demonstrated that the PD-1 expression balance between Treg and Teff cells could determine the clinical efficacy of PD-1 blockade therapies (41). In order to selectively make various immunotherapies on the targeted lymphocyte subset and obtain the optimum clinical benefits, it will be more important to develop biomarkers that accurately evaluate the immune status of each patient. We previously demonstrated that chronic GVHD is characterized by constitutive phosphorylation of STAT5 in Tcons associated with elevated amounts of IL-7 and IL-15 and relative functional deficiency of IL-2 (17). IL-2 therapy resulted in the selective increase of STAT5 phosphorylation in Tregs and the decrease of phosphorylated STAT5 in Tcons (17). Thus, as well as CD25 expression mentioned above, STAT5 phosphorylation could be a candidate as a biomarker to predict the response of each T cell subset to exogenous IL-2, however, further studies are warranted for the application to the clinical settings.

This study has several limitations. First, all experiments in this study were performed using only one experimental transplant system. Given the great variety of clinical transplants, the validation experiments using the second GVHD model system are important to consider the clinical relevance. Second, the experimental system for assessing the GVL effect in this study is not completely relevant to the clinical setting. We utilized the P815-mastocytoma tumor model, however, the majority of the HSCTs are indicated to treat patients with acute myeloid leukemia (AML). Therefore, it should be validated the hypothesis of this work in such myeloid malignancies as the MLL-AF9 AML model in the future. Third, the experimental system used in this study could not evaluate the role of NK cells in the GVL effect increased by IL-2 therapy. A previous study analyzed samples from patients and demonstrated that low-dose IL-2 activates NK cells as well as Tregs (25). We confirmed that IL-2 administration to non-transplanted normal mice increased NK cells in an IL-2 dose-dependent manner, however, IL-2 administration to allo-transplanted recipient mice did not increase NK cells in the experimental HSCT used in this study (**Supplemental Figure S4**). In fact, previous studies have shown that more than 50 million spleen cells need to be infused at transplant in order to observe NK cells in the B6-into-B6D2F1 system (42, 43). In our experimental setting, we infused 5 million spleen cells and it appears to be not enough to evaluate NK cells after transplant. The effect of NK cells on the GVL effect after IL-2 therapy should be tested by the experiments with the other settings, which may contribute to identifying the responsible cells for the anti-tumor activity increased by IL-2 therapy.

In conclusion, our data suggested that the responses of Tregs and effector T cells to exogenous IL-2 differs depending on the immune environment in the host, and the mutual balance of the response between the subsets has a substantial impact on the modulating effect of GVHD and GVL after IL-2 initiation. Our findings may provide useful information to develop personalized IL-2 therapy for each patient having different immune status.

## Methods

### Mice

Female C57BL/6J (B6, H-2Kb) and B6D2F1 (H-2Kb/d) mice were purchased from Japan SLC (Hamamatsu, Japan).

### Bone marrow transplantation

Following standard protocols (44), B6D2F1 mice received lethal X-ray total body irradiation in two doses, and injected on day 0 with 5 × 10^6^ spleen cells and 5 × 10^6^ T cell-depleted bone marrow from B6 (H-2Kb, Ly-5.1) mice. The radiation dose for the two doses was 10 Gy in all experiments except for in **Figure 5**. In **Figure 5**, we conducted the experiment in the settings with TBI 13 Gy in addition to TBI 10 Gy. To assess GVL effects, 1 × 10^5^ or 2.5 × 10^4^ luciferase P815 cells were intravenously injected along with 1 × 10^6^ or 5 × 10^6^ donor spleen cells or T cell-depleted bone marrow. P815 (H-2Kd) is a mastocytoma derived from a DBA/2 mouse. T cells were depleted or purified using anti-CD90.2 Microbeads on an AutoMACS system (Miltenyi Biotec, Auburn, CA), following the manufacturer’s instructions. Mice were subcutaneously administered with 50, 500, 1,000, 5,000, 20,000, 40,000 or 100,000 IU/mouse of recombinant human IL-2 (Teceleukin; Shionogi & Co., Ltd.) in sterile 200 µl PBS.

### Assessment of GVHD and GVL effect

Survival after bone marrow transplantation was monitored daily, and clinical GVHD was assessed 2-3 times a week using a scoring system (maximum score 10) based on weight loss, posture, activity, fur texture, and skin integrity, as described previously (45, 46). Bioluminescence from tumor cells expressing luciferase was measured weekly to quantify tumor burden. Briefly, mice were injected with 3 mg/mouse D-luciferin (OZ Biosciences, Marseille, France), anesthetized 10 minutes thereafter, and imaged on an IVIS Spectrum imaging system (Caliper Life Sciences, Hopkinton, MA) to measure total flux in photons/sec.

### Antibodies and flow cytometry

PE- and eFluor450-conjugated anti-CD4 (GK1.5), PerCP/Cy5.5- and APC/eFlour780-conjugated anti-CD8 (53-6.7), PE-conjugated anti-CD25 (PC61.5), PE-conjugated anti-Ki67 (SolA15), PE-conjugated anti-CTLA-4 (UC10-4B9), PE-conjugated anti-Tim-3 (RMT3-23), PE/Cyanine7- and APC/eFlour780-conjugated anti-CD44 (MI7), FITC-conjugated anti-CD62L (MEL-14), PE/Cyanine5-conjugated anti-ICOS (7E.17G9), PerCP/Cyanine5.5-conjugated anti-CD45.1 (A20), PE/Cyanine7-conjugated anti-GITR (DTA-1), FITC-conjugated anti-LAG-3 (eBioC9B7W), PE-conjugated anti-PD-1 (RMP1-30), and APC-conjugated anti-Foxp3 (FJK-16s) were obtained from eBioscience (San Diego, CA). FITC-conjugated anti-H-2Kd (SF1-1.1) and FITC-conjugated anti-Stat5 (pY694) were obtained from BD Bioscience. PE/Cyanine7-conjugated anti-CCR4 (2G12) and PE/Cyanine7-conjugated anti-CCR7 (4G12) was obtained from Biolegend. Intracellular FoxP3, Ki-67, and Stat5 were stained with an anti-mouse/rat Foxp3 staining kit (eBioscience, San Diego, CA). Cells were stained in phosphate-buffered saline containing 2% fetal calf serum, and sorted on a MACSQuant system with MACSQuantify software (Miltenyi Biotec, Bergisch Gladbach, Germany). Data were analyzed in FlowJo (Treestar, Ashland, OR)

### *In vivo* proliferation and *in vitro* suppression assay

CD4^+^CD25^+^ Tregs and CD4^+^CD25^-^ Tcons were isolated from murine spleen cells on a FACS Aria. Tcons from naïve B6 mice were labeled with CellTraceTM Violet according to the manufacturer’s protocols. 5 × 10^4^ Tcons were cultured in the wells of a 96-well plate together with various concentrations of Tregs and 2.5 × 10^4^ irradiated (20 Gy) peritoneal cells in the presence of with 5 μg/mL platebound anti-CD3ϵ mAbs. CD62L^+^ and CD62L^-^ T cells were isolated from splenocytes obtained from CD45.1 mice using an autoMACS Pro Separator (Miltenyi Biotec), Pan T Cell Negative Selection Kit, and CD62L Isolation Kit. These cells were also labeled with CellTrace™ Violet according to the manufacturer’s protocols, and adoptively transplanted to recipient mice. Proliferation was analyzed after sorting to > 97% purity on a MACSQuant flow cytometer.

### Statistics

Results are reported as mean +/- SEM. Student’s t-test and ANOVA with Bonferroni’s correction were used to compare two and > 2 groups in Prism version 5.0 (GraphPad Software, San Diego, CA), with p < 0.05 considered statistically significant. Log-rank test was used to assess survival.

### Study approval

All animal experiments were compliant with regulations of the Institutional Animal Care and Research Advisory Committee, Okayama University Advanced Science Research Center.

## Data availability statement

The original contributions presented in the study are included in the article/**Supplementary Material**. Further inquiries can be directed to the corresponding author.

## Ethics statement

The animal study was reviewed and approved by Institutional Animal Care and Research Advisory Committee, Okayama University Advanced Science Research Center.

## Author contributions

YuM designed and performed experiments and wrote the paper. TA, TY, YK, MI, MN, YaS, HS, SI, TK, YuS, and YoM performed experiments and edited the paper. KM supervised the laboratory studies and edited the paper. All authors contributed to the article and approved the submitted version.

## Funding

This work was supported by Japan Society for the Promotion of Science KAKENHI Grant No. 20K08753.

## Acknowledgments

The authors thank all staffs at Institutional Animal Care and Research Advisory Committee, Okayama University Advanced Science Research Center and at Central Research Laboratory, Okayama University Medical School.

## Conflict of interest

The authors declare that the research was conducted in the absence of any commercial or financial relationships that could be construed as a potential conflict of interest.

## Publisher’s note

All claims expressed in this article are solely those of the authors and do not necessarily represent those of their affiliated organizations, or those of the publisher, the editors and the reviewers. Any product that may be evaluated in this article, or claim that may be made by its manufacturer, is not guaranteed or endorsed by the publisher.

## References

[1] Sakaguchi S Sakaguchi N Asano M Itoh M Toda M. Immunologic self-tolerance maintained by activated T cells expressing IL-2 receptor alpha-chains (CD25). breakdown of a single mechanism of self-tolerance causes various autoimmune diseases. J Immunol (1995) 155:1151–64. 7636184

[2] Fontenot JD Rasmussen JP Williams LM Dooley JL Farr AG Rudensky AY. Regulatory T cell lineage specification by the forkhead transcription factor foxp3. Immunity (2005) 22:329–41. doi: 10.1016/j.immuni.2005.01.016 15780990

[3] Fontenot JD Gavin MA Rudensky AY. Foxp3 programs the development and function of CD4+CD25+ regulatory T cells. Nat Immunol (2003) 4:330–6. doi: 10.1038/ni904 12612578

[4] Hori S Nomura T Sakaguchi S. Control of regulatory T cell development by the transcription factor Foxp3. Sci (80- ) (2003) 299:1057–61. doi: 10.1126/science.1079490 12522256

[5] Hoffmann P Ermann J Edinger M Fathman CG Strober S. Donor-type CD4(+)CD25(+) regulatory T cells suppress lethal acute graft-versus-host disease after allogeneic bone marrow transplantation. J Exp Med (2002) 196:389–99. doi: 10.1084/jem.20020399 PMC2193938 12163567

[6] Taylor PA Lees CJ Blazar BR. The infusion of ex vivo activated and expanded CD4(+)CD25(+) immune regulatory cells inhibits graft-versus-host disease lethality. Blood (2002) 99:3493–9. doi: 10.1182/blood.V99.10.3493 11986199

[7] Edinger M Hoffmann P Ermann J Drago K Fathman CG Strober S. CD4+CD25+ regulatory T cells preserve graft-versus-tumor activity while inhibiting graft-versus-host disease after bone marrow transplantation. Nat Med (2003) 9:1144–50. doi: 10.1038/nm915 12925844

[8] Taylor PA Panoskaltsis-Mortari A Swedin JM Lucas PJ Gress RE Levine BL. L-selectin(hi) but not the l-selectin(lo) CD4+25+ T-regulatory cells are potent inhibitors of GVHD and BM graft rejection. Blood (2004) 104:3804–12. doi: 10.1182/blood-2004-05-1850 15292057

[9] Ermann J Hoffmann P Edinger M Dutt S Blankenberg FG Higgins JP. Only the CD62L+ subpopulation of CD4+CD25+ regulatory T cells protects from lethal acute GVHD. Blood (2005) 105:2220–6. doi: 10.1182/blood-2004-05-2044 15546950

[10] Hoffmann P Eder R Boeld TJ Doser K Piseshka B Andreesen R. Only the CD45RA+ subpopulation of CD4+CD25high T cells gives rise to homogeneous regulatory T-cell lines upon *in vitro* expansion. Blood (2006) 108:4260–7. doi: 10.1182/blood-2006-06-027409 16917003

[11] Sykes M Romick ML Hoyles KA Sachs DH. *In vivo* administration of interleukin 2 plus T cell-depleted syngeneic marrow prevents graft-versus-host disease mortality and permits alloengraftment. J Exp Med (1990) 171:645–58. doi: 10.1084/jem.171.3.645 PMC2187782 2307931

[12] Sykes M Abraham VS Harty MW Pearson DA. IL-2 reduces graft-versus-host disease and preserves a graft-versus-leukemia effect by selectively inhibiting CD4+ T cell activity. J Immunol (1993) 150:197–205. 8093257

[13] Sykes M Harty MW Szot GL Pearson DA. Interleukin-2 inhibits graft-versus-host disease-promoting activity of CD4+ cells while preserving CD4- and CD8-mediated graft-versus-leukemia effects. Blood (1994) 83:2560–9. doi: 10.1182/blood.V83.9.2560.2560 7909457

[14] Soiffer RJ Murray C Gonin R Ritz J. Effect of low-dose interleukin-2 on disease relapse after T-cell-depleted allogeneic bone marrow transplantation. Blood (1994) 84:964–71. doi: 10.1182/blood.V84.3.964.964 8043878

[15] Soiffer RJ Murray C Cochran K Cameron C Wang E Schow PW. Clinical and immunologic effects of prolonged infusion of low-dose recombinant interleukin-2 after autologous and T-cell-depleted allogeneic bone marrow transplantation. Blood (1992) 79:517–26. doi: 10.1182/blood.V79.2.517.517 1730094

[16] Koreth J Matsuoka K Kim HT McDonough SM Bindra B Alyea 3EP. Interleukin-2 and regulatory T cells in graft-versus-host disease. N Engl J Med (2011) 365:2055–66. doi: 10.1056/NEJMoa1108188 PMC3727432 22129252

[17] Matsuoka KI Koreth J Kim HT Bascug G McDonough S Kawano Y. Low-dose interleukin-2 therapy restores regulatory T cell homeostasis in patients with chronic graft-versus-host disease. Sci Transl Med (2013) 5 :179ra43. doi: 10.1126/scitranslmed.3005265 23552371 PMC3686517

[18] Setoguchi R Hori S Takahashi T Sakaguchi S. Homeostatic maintenance of natural Foxp3(+) CD25(+) CD4(+) regulatory T cells by interleukin (IL)-2 and induction of autoimmune disease by IL-2 neutralization. J Exp Med (2005) 201:723–35. doi: 10.1084/jem.20041982 PMC2212841 15753206

[19] Boyman O Sprent J. The role of interleukin-2 during homeostasis and activation of the immune system. Nat Rev Immunol (2012) 12:180–90. doi: 10.1038/nri3156 22343569

[20] Asano T Meguri Y Yoshioka T Kishi Y Iwamoto M Nakamura M. PD-1 modulates regulatory T-cell homeostasis during low-dose interleukin-2 therapy. Blood (2017) 129:2186–97. doi: 10.1182/blood-2016-09-741629 PMC5391624 28151427

[21] Ikegawa S Matsuoka KI. Harnessing treg homeostasis to optimize posttransplant immunity: Current concepts and future perspectives. Front Immunol (2021) 12:713358. doi: 10.3389/fimmu.2021.713358 34526990 PMC8435715

[22] Koreth J Kim HT Jones KT Lange PB Reynolds CG Chammas MJ. Efficacy, durability, and response predictors of low-dose interleukin-2 therapy for chronic graft-versus-host disease. Blood (2016) 128:130–7. doi: 10.1182/blood-2016-02-702852 PMC4937358 27073224

[23] Matsuoka KI. Low-dose interleukin-2 as a modulator of treg homeostasis after HSCT: current understanding and future perspectives. Int J Hematol (2018) 107:130–7. doi: 10.1007/s12185-017-2386-y 29234980

[24] Whangbo JS Kim HT Mirkovic N Leonard L Poryanda S Silverstein S. Dose-escalated interleukin-2 therapy for refractory chronic graft-versus-host disease in adults and children. Blood Adv (2019) 3:2550–61. doi: 10.1182/bloodadvances.2019000631 PMC6737411 31471324

[25] Hirakawa M Matos T Liu H Koreth J Kim HT Paul NE. Low-dose IL-2 selectively activates subsets of CD4+ tregs and NK cells. JCI Insight (2016) 1:1–18. doi: 10.1172/jci.insight.89278 PMC5085610 27812545

[26] Qu Y Zhang B Liu S Zhang A Wu T Zhao Y. 2-gy whole-body irradiation significantly alters the balance of CD4 +CD25-T effector cells and CD4+CD25 +Foxp3+ T regulatory cells in mice. Cell Mol Immunol (2010) 7:419–27. doi: 10.1038/cmi.2010.45 PMC4002961 20871628

[27] Bayer AL Jones M Chirinos J De Armas L Schreiber TH Malek TR. Host CD4+CD25+ T cells can expand and comprise a major component of the treg compartment after experimental HCT. Blood (2009) 113:733–43. doi: 10.1182/blood-2008-08-173179 PMC2628379 18832651

[28] Shatry A Levy RB. *In situ* activation and expansion of host tregs: A new approach to enhance donor chimerism and stable engraftment in MHC- matched allogeneic HCT. Biol Blood Marrow Transplant (2010) 15:785–94. doi: 10.1016/j.bbmt.2009.03.011.In PMC2700954 19539209

[29] Anderson BE Taylor PA McNiff JM Jain D Demetris AJ Panoskaltsis-Mortari A. Effects of donor T-cell trafficking and priming site on graft-versus-host disease induction by naive and memory phenotype CD4 T cells. Blood (2008) 111:5242–51. doi: 10.1182/blood-2007-09-107953 PMC2384145 18285547

[30] Foster AE Marangolo M Sartor MM Alexander SI Hu M Bradstock KF. Human CD62L- memory T cells are less responsive to alloantigen stimulation than CD62L+ naive T cells: potential for adoptive immunotherapy and allodepletion. Blood (2004) 104:2403–9. doi: 10.1182/blood-2003-12-4431 15231569

[31] Anderson BE McNiff J Yan J Doyle H Mamula M Shlomchik MJ. Memory CD4+ T cells do not induce graft-versus-host disease. J Clin Invest (2003) 112:101–8. doi: 10.1172/JCI17601 PMC162285 12840064

[32] Nguyen VH Zeiser R Daniel L Chang DS Beilhack A Contag CH. *In vivo* dynamics of regulatory T-cell trafficking and survival predict effective strategies to control graft-versus-host disease following allogeneic transplantation. Blood (2007) 109:2649–56. doi: 10.1182/blood-2006-08-044529 17095616

[33] Buxbaum NP Farthing DE Maglakelidze N Lizak M Merkle H Carpenter AC. *In vivo* kinetics and nonradioactive imaging of rapidly proliferating cells in graft-versus-host disease. JCI Insight (2017) 2:e92851. doi: 10.1172/jci.insight.92851 PMC5470940 28614804

[34] Tomura M Honda T Tanizaki H Otsuka A Egawa G Tokura Y. Activated regulatory T cells are the major T cell type emigrating from the skin during a cutaneous immune response in mice. J Clin Invest (2010) 120:883–93. doi: 10.1172/JCI40926 PMC2827959 20179354

[35] Lu SY Liu KY Liu DH Xu LP Huang XJ. High frequencies of CD62L(+) naive regulatory T cells in allografts are associated with a low risk of acute graft-versus-host disease following unmanipulated allogeneic haematopoietic stem cell transplantation. Clin Exp Immunol (2011) 165:264–77. doi: 10.1111/j.1365-2249.2011.04418.x PMC3142651 21635226

[36] Zeiser R Blazar BR. Pathophysiology of chronic graft-versus-Host disease and therapeutic targets. N Engl J Med (2017) 377:2565–79. doi: 10.1056/nejmra1703472 29281578

[37] Luznik L O’Donnell PV Fuchs Ephraim J. Post-transplantation cyclophosphamide for tolerance induction in HLA-haploidentical BMT graft failure, graft-versus-host disease, and infection: the devil’s triangle. Semin Oncol (2013) 39:1–16. doi: 10.1053/j.seminoncol.2012.09.005.Post-transplantation PMC3808078 23206845

[38] Mehta RS Holtan SG Wang T Hemmer MT Spellman SR Arora M. Composite GRFS and CRFS outcomes after adult alternative donor HCT. J Clin Oncol (2020) 38:2062–76. doi: 10.1200/JCO.19.00396 PMC7302955 32364845

[39] Grunwald MR Zhang MJ Elmariah H Johnson MH Martin AS Bashey A. Alternative donor transplantation for myelodysplastic syndromes: Haploidentical relative and matched unrelated donors. Blood Adv (2021) 5:975–83. doi: 10.1182/bloodadvances.2020003654 PMC7903230 33576783

[40] Seddiki N Santner-Nanan B Martinson J Zaunders J Sasson S Landay A. Expression of interleukin (IL)-2 and IL-7 receptors discriminates between human regulatory and activated T cells. J Exp Med (2006) 203:1693–700. doi: 10.1084/jem.20060468 PMC2118333 16818676

[41] Kumagai S Togashi Y Kamada T Sugiyama E Nishinakamura H Takeuchi Y. The PD-1 expression balance between effector and regulatory T cells predicts the clinical efficacy of PD-1 blockade therapies. Nat Immunol (2020) 21:1346–58. doi: 10.1038/s41590-020-0769-3 32868929

[42] Pattengale PK Ramstedt U Gidlund M Örn A Axberg I Wigzell H. Natural killer activity in (C57BL/6 × DBA/2)F1 hybrids undergoing acute and chronic graft-vs.-host reaction. Eur J Immunol (1983) 13:912–9. doi: 10.1002/eji.1830131110 6641788

[43] Roy C Ghayur T Kongshavn PA Lapp WS. Natural killer activity by spleen, lymph node, and thymus cells during the graft-versus-host reaction. Transplantation (1982) 34:144–6. doi: 10.1097/00007890-198209000-00006 7135468

[44] Teshima T Ordemann R Reddy P Gagin S Liu C Cooke KR. Acute graft-versus-host disease does not require alloantigen expression on host epithelium. Nat Med (2002) 8:575–81. doi: 10.1038/nm0602-575 12042807

[45] Matsuoka K Ichinohe T Hashimoto D Asakura S Tanimoto M Teshima T. Fetal tolerance to maternal antigens improves the outcome of allogeneic bone marrow transplantation by a CD4+ CD25+ T-cell-dependent mechanism. Blood (2006) 107:404–9. doi: 10.1182/blood-2005-07-3045 16150938

[46] Cooke KR Kobzik L Martin TR Brewer J Delmonte J Crawford JM. An experimental model of idiopathic pneumonia syndrome after bone marrow transplantation: I. the roles of minor h antigens and endotoxin. Blood (1996) 88:3230–9. doi: 10.1182/blood.V88.8.3230.bloodjournal8883230 8963063

