# Supplementary material for: Responses of regulatory and effector T-cells to low-dose interleukin-2 differ depending on the immune environment after allogeneic stem cell transplantation
Source: Front Immunol. 2022 Aug 2;13:891925. doi: 10.3389/fimmu.2022.891925 (PMC9379320; doi:10.3389/fimmu.2022.891925)
Supplement: Supplementary file 1 [file DataSheet_1.pdf]

Supplementary Materials for

Responses of regulatory and effector T-cells to low-dose interleukin-2 differ depending on the immune environment after allogeneic stem cell transplantation

Yusuke Meguri, Takeru Asano, Takanori Yoshioka, Miki Iwamoto, Shyuntaro Ikegawa, Hiroyuki Sugiura, Yuriko Kishi, Makoto Nakamura, Yasuhisa Sando, Takumi Kondo, Yuichi Sumii, Yoshinobu Maeda, and Ken-ichi Matsuoka

Department of Hematology and Oncology, Okayama University Graduate School of Medicine, Dentistry and Pharmaceutical Sciences, Okayama, Japan

Address correspondence to Ken-ichi Matsuoka, M.D., Ph.D.

Department of Hematology and Oncology, Okayama University, 2-5-1 Shikata-cho, Kita-ku, Okayama 700-8558, Okayama, Japan.

Phone: +81-86-235-7227; Fax: +81-86-232-8226

E-mail: k-matsu@md.okayama-u.ac.jp.

Supplemental Figure 1.

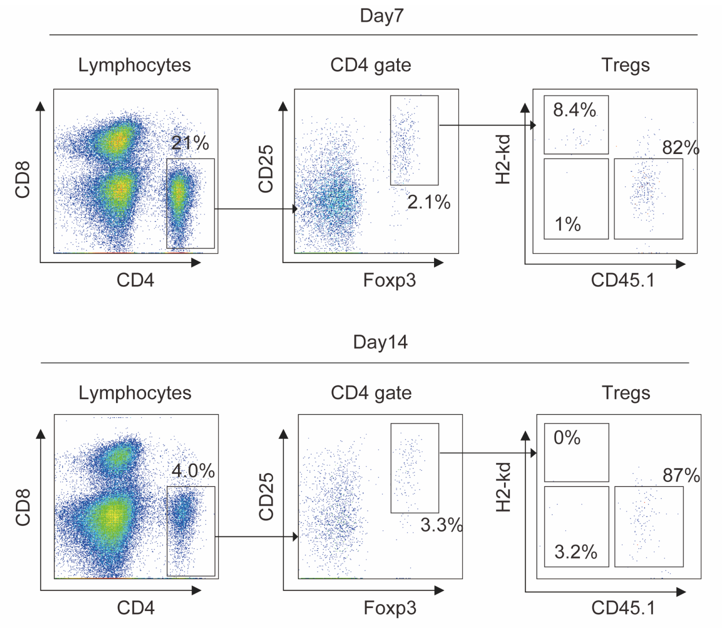

Host Treg remains on day 7 after HSCT in the transplantation setting in this study.

Irradiated B6D2F1 mice were injected on day 0 with 5×10^6 spleen cells and 5×10^6 T cell-depleted bone marrow from B6 mice. we sacrificed mice and analyzed chimerism of each T cell subset in spleen on day 7, 14, 21, 28, and 35. Representative FACS gating strategy to analyze the chimerism of Treg on day 7 and 14 are shown. H2-kd$^+$CD45.1$^-$ host-type Tregs still remained on day 7 and they disappeared on day 14.

Supplemental Figure 2.

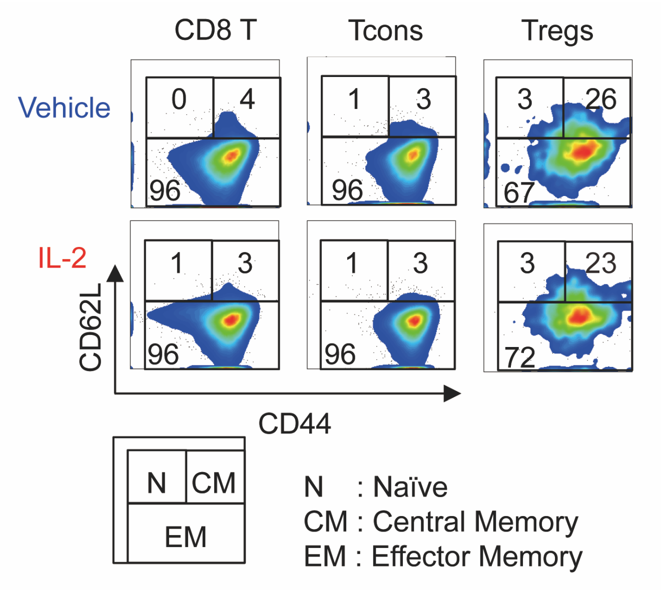

Representative figure of lymphocyte subsets at week 2

Lethally irradiated B6D2F1 mice were transplanted with 5×10^6 spleen cells and 5×10^6 bone marrow cells from donor B6 mice, and vehicle or low-dose IL-2 were subcutaneously administrated once per day for 15 days. Spleen cells were analyzed at week2 after transplantation. Representative figures to identify $CD44^{low}CD62L^{high}$ naive (N), $CD44^{high}CD62L^{high}$ central-memory (CM), and $CD44^{high}CD62L^{low}$ effector-memory (EM) subsets within CD8 T cells, Tcons, and Tregs at week 2. Upper and lower panels are representative of mice treated with vehicle and IL-2, respectively.

Supplemental Figure 3.

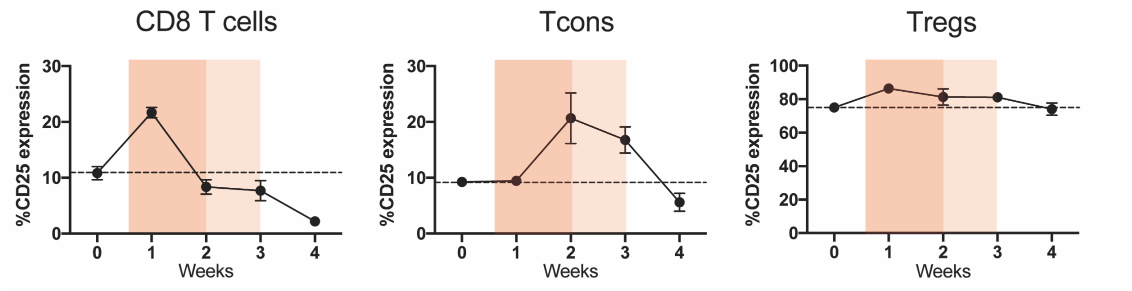

The changes in the frequency of CD25 positive cells of each subset over time post-transplant

Lethally irradiated (10 Gy) B6D2F1 mice received 5×10^6 CD45.2+ TCD-BM and 5×10^6 CD45.1+ spleen cells from B6 donor mice. Post-transplant treatments with IL-2 or vehicle were not administrated in this experiment. Percentage of CD25 positive cells of CD8 T cells, Tcons, and Tregs from week 0 to week 4 after transplantation are shown. Thin and thick orange shades indicate the two different phases of clinical GVHD. The dash lines represent the baseline at week 0. Bars show mean +/- SEM.

Supplemental Figure 4.

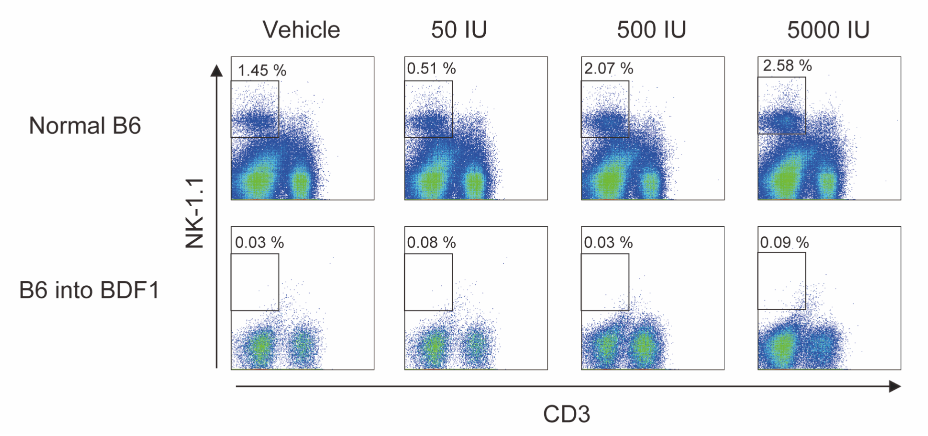

Response of NK cells after IL-2 treatment in non-transplanted mice and allo-transplanted mice

Irradiated B6D2F1 mice were injected on day 0 with 5×10^6 spleen cells and 5×10^6 T cell-depleted bone marrow from B6 mice. Then, recipient B6D2F1 mice were subcutaneously administered with 50, 500, or 5,000 IU/mouse of recombinant human IL-2 in sterile 200 µl PBS, every day from day 5 to 14. As a control, normal B6 mice were administered with IL-2 as same as transplanted recipient mice for 10 days. After IL-2 treatment, we sacrificed mice and analyzed $NK1.1^+CD3^-$ NK cells in spleen.
